# Why open-access publication should be nonprofit—a view from the field of theoretical language science

**DOI:** 10.3389/fnbeh.2013.00057

**Published:** 2013-06-06

**Authors:** Martin Haspelmath

**Affiliations:** Department of Linguistics, Max Planck Institute for Evolutionary AnthropologyLeipzig, Germany

Many of my fellow theoretical linguistics researchers have not noticed the momentous changes in the world of science publication yet. When confronted with the idea that publication costs should be covered by author fees (“author processing charges,” or APCs), they often react with disbelief and indignation.

But the signs of inefficiency of the old subscription-based system are just as clear in my field as elsewhere, so I see no reasonable alternative to Gold open access (i.e., freely accessible electronic publications on the publisher's website). Green open access is inefficient because of the duplication of efforts, and subscription is inefficient because it is very difficult to predict for an institution to what extent its members will want to use a journal or book. Moreover, the subscription-based model is even worse for scholars with low budgets: While a low-budget scholar can at least read the richer scholars' works on the APC-based open access model, not even that is possible on the traditional model, and usually one can publish in prestigious places only if one knows the relevant literature.

But is APC-based publication of scientific results by profit-oriented companies (such as Macmillan Publishers, which owns Nature Publishing Group, the partner of Frontiers) a good alternative to subscription? Clearly, the old author-pays model removes a major inefficiency of the subscription-based system, because the authors know that they want to publish, whereas the subscribers only suspect that they want to use the publications. According to Stuart Shieber, an open-access expert and theoretical linguist at Harvard University, subscription-based publication can lead to market dysfunction (unreasonably high publication prices) because science journals are not competitive goods: If you subscribe to one science journal, this doesn't mean that you don't need another one (see Shieber, 2013). But from the author's perspective, Shieber says, they are competitive goods: You just need to publish in one journal, and you can choose the cheapest one.

Shieber's article is very sophisticated from an economics perspective, but it completely leaves aside a crucial component of scientific publication that I will argue leads to market dysfunction also with the APC-based open-access model: Scientific publications serve both to disseminate research results and to build careers of scientists. The success of a scientist (and of groups of scientists) is routinely measured by the place of publication of the work. When evaluating a scientist, the evaluators not only look at the amount of research output and the amount of citations, but also at the place of publication. Moreover, when deciding what to cite, scientists routinely privilege papers published in more prestigious journals and books published in more prestigious imprints. Thus, to be a successful scientist, one needs to publish in the same places as other successful scientists. Thus, journals and imprints have a significance for science that goes far beyond the purpose of dissemination of research results. The latter can nowadays be achieved much more easily, by archives such as Arxiv.org, or by publishing in one's personal blog, or on Academia.edu. The primary purpose of peer review is actually peer selection: One needs to make a special effort to present one's results in such a way that one's peers recognize their value. It is only in this way that one's research is likely to have an impact on others. Being selected for publication in a particular place (journal or book imprint) means being successful.

One could imagine alternative models of establishing scientific credentials, e.g., by a rating system similar to the one found in online bookshops, but discussing these is beyond the scope of this note. The big advantage of anonymous peer review and selection that I see for my own field is that it gives younger scholars the chance to become more widely visible even without traveling to many conferences. In the following, I assume that peer selection of publications will be the prevalent mode of establishing scientific credentials also in the future.

Now crucially, the association of place of publication with prestige means that the market for APC-based journals does NOT provide for competition after all: I cannot simply submit my paper to a cheaper journal if the cheaper journal has much less prestige and will lead to much fewer citations of the article. I will quite likely submit my paper to the best journal in my subfield even if this means that I will pay higher APCs (as long as my budget still allows it). Publishers will be able to price their journals according to their prestige, not according to their services. But in the 21st century, the prestige of a journal is primarily the result of the work of the scientists who publish in it, who serve as editors and as reviewers, and not the result of the publisher's efforts. If I publish an excellent piece of research in a journal, or if I write a careful review of a submitted manuscript, I thereby enhance the prestige of this journal, and I thereby contribute to making the journal more expensive for future submitters. The publishers will reap the benefits of my excellent and conscientious work, because they can charge more without improving their services. This situation is clearly undesirable for science.

Journal and book publication has become very simple and cheap as a result of technological developments: One just needs typesetting, hosting and web presentation, as well as perhaps some kind of print-on-demand service (for open-access books). This can be done very easily without major investments, and as a result, journal publication in the less wealthy countries has increased dramatically over the last 20 years. For example, the Brazilian platform Scielo.org hosts over 1000 journals that are freely accessible and do not charge any author fees.

Of course, even nowadays journal and book publication does not come for free, and somebody has to pay for it. But in order to have a functioning market with reasonable prices, one needs real competition. My research institution can replace its cleaning company by another one, or it can buy its computers and printers from different companies if we are dissatisfied with the services and products. But we cannot simply replace journals and imprints, because we use these to build our careers and to measure our success.

A functioning model would be one where the scientists own the journal titles and book imprints, and where they choose typesetters, webdesign companies, and hosting companies that can be easily replaced by others if the prices are not right. Just as basic science itself is not a profit-oriented activity, publication of scientific results would not be a profit-oriented activity. APCs could be charged by the nonprofit organizations of the scientists (universities, scientific libraries, scholarly associations), but these would not increase as a result of excellent and high-impact work being published by the journals and imprints. On the contrary, since universities and scholarly associations derive their prestige in part from their publications, it is to be expected that the best work will be published without any APCs: These nonprofit organizations would benefit from their prestigious journals and imprints, so it would make sense for them to subsidize them in much the same way as they are subsidizing non-profit-oriented basic research itself.

The alternative model, where APCs are charged by profit-oriented publishers, has another serious drawback: It creates a strong incentive to create journals and book imprints that function like “vanity presses,” allowing authors to publish their low-quality work without significant risk of rejection. Vanity presses have long existed in the regular book market, and they have not been a problem because no public money went into them. Of course, everyone should be free to publish their bad novels or low-quality scientific articles if they desire. However, when it comes to scientific publications, the idea is that the APCs are covered by grants for scientific research, i.e., mostly by public money that would otherwise go into science. In the traditional system, grant holders are free to publish the results of their research wherever they want—but there used to be a limited set of possibilities, and scientific vanity publishers hardly existed. But nowadays increasingly, grant agencies are trying to impose the restriction that the publication should be open access—and with the for-profit approach, there is an unlimited set of possibilities. Anyone can easily found a new journal and offer publication for APCs, simply claiming that it is peer-reviewed. For example, I recently heard of two Chinese companies that are publishing a large number of open-access journals, some of them in my field of linguistics: Wuhan-based SCIRP (http://www.scirp.org/, over 250 journals) and Beijing-based MDPI (http://www.mdpi.com/, over 120 journals). The business model here is to start a large number of new journals and to hope that some of them will succeed and bring profit. For example, MDPI's journal *Languages* does not even have an editor yet. This is of course reminiscent of the business model of spam e-mail, and in fact, some observers have warned of the danger of “predatory journals.” In particular, Jeffrey Beal noted in a *Nature* column in 2012 that there are hundreds of journals with this business model, and he writes:

The competition for author fees among fraudulent publishers is a serious threat to the future of science communication. To compete in a crowded market, legitimate open-access publishers are being forced to promise shorter submission-to-publication times; this weakens the peer-review process, which takes time to do properly. To tackle the problem, scholars must resist the temptation to publish quickly and easily… (Beall, 2012)

But the problem with Beall's argumentation is that it is difficult to say in what sense the business model of “predatory” publishers is “fraudulent.” They are just exploiting a new niche that has been created by the notion that authors should pay for publication by profit-oriented companies. Clearly, given the current system, where not only quality, but also quantity of publication counts, scholars have an incentive to publish “quickly and easily.” Moral exhortations to “resist the temptation” will not make this problem go away.

In order to prevent scholars from publishing their work in less than fully respectable venues, science funders will have to set up a new control system that monitors journal publishers and that prevents grant holders from using grant money to publish in these journals. It is difficult to see how this can be done efficiently and without unduly restricting the freedom of scientists. In any event, it will cost money that would be saved if publication costs were carried by the publishers (universities, libraries, scholarly associations), rather than by the authors.

Another argument that Shieber (2013) cites against toll-access publication is that traditional publishers typically use price bundling, so that canceling individual journal subscriptions does not significantly reduce the costs of the libraries. But is this different in the for-profit open-access model? Not at all: Once open-access publication becomes the norm, for-profit publishers will introduce price bundling for APCs: If your institution enters into an agreement with the publisher, you will pay only EUR 500 for publishing your paper instead of the usual EUR 1000. There are already signs that this is happening: In January 2013, De Gruyter and the Max Planck Society came to an agreement about open-access publication of Max Planck books by De Gruyter (see http://www.mpdl.mpg.de/news/pressrel_2013/PM_deGruyter_MPG_de.pdf).

To summarize, the major argument for open access is that toll access is inefficient because there can be no functioning market (Shieber, 2013) and because it is difficult for subscribers to predict their needs. How should open-access publication be funded? One common funding option is by public funds, i.e., publication is funded in the same way in which science is funded. The other major funding option is by for-profit companies, on the basis of APCs. The major argument against for-profit companies is again that there can be no functioning market: Scientific publications not only serve to disseminate research findings, but they also build scientific prestige and reputation. Thus, they should be owned by scientists and their institutions, not by companies whose main purpose is to make money. If scientific work is published by for-profit companies, they make money from the reputation that is built up by publicly-funded scientific work. This means that scientific work should be published by nonprofit organizations—those very organizations that are engaged in doing science. This is in fact the traditional model of the 19th century, when it was primarily the scholarly societies and academies that published scientific works. It turns out that this is also the best model for the future.
